# Do DNA Repair Gene Mutations Cause Clonal Hematopoiesis?

**DOI:** 10.3390/cancers18040691

**Published:** 2026-02-20

**Authors:** Dik C. van Gent, Zofia M. Komar

**Affiliations:** Department of Molecular Genetics, Erasmus Medical Center Cancer Institute, University Medical Center, 3015 CN Rotterdam, The Netherlands

The maintenance of genome integrity by DNA damage response (DDR) mechanisms is indispensable to sustain life. Repair of DNA lesions has been observed in all known organisms from bacteria to humans. The complexity and the number of genetic pathways involved in preserving genome function have increased, especially in multicellular organisms. In addition to the repair of DNA damage, regulation of the cell cycle (to prevent replication or mitosis when the DNA is still damaged) and apoptosis (to eliminate cells that contain too much DNA damage) are also required to counteract cancer and aging. These mechanisms are collectively called the DNA damage response (DDR). Depending on the exact DDR pathway that is compromised, several types of hereditary cancer syndromes can develop in patients. One of the best-known cancer predisposition syndromes is hereditary cancer caused by mutations in the BRCA1 or BRCA2 genes. Women that are heterozygous for deleterious mutations in one of these genes have a severely increased risk of developing breast or ovarian cancer. However, the chance to develop prostate cancer in men and pancreatic cancer in both men and women is also elevated in these mutation carriers.

In a recent publication, Marshall et al [1] examined whether these mutation carriers are also at increased risk of developing hematologic aberrations (Figure 1). They investigated the occurrence of the outgrowth of white blood cell clones derived from a single progenitor cell (clonal hematopoiesis or CH). This is a relatively common phenomenon in older people and may be enhanced by DDR defects [2]. Such clones often carry mutations in various genes that may have given them a selective advantage. For example, mutations in the TP53 gene give cells a growth advantage because of reduced apoptosis, or DNMT3A mutations may result in deregulated gene expression [3]. These clonal outgrowths are benign and do not result in tumor formation in the large majority of cases. However, they contain mutations that can also be found in leukemia, suggesting that CH clones may develop into this disease. Therefore, increased CH is a risk factor for leukemia development [4]. CH can be defined as the occurrence of pathogenic or likely pathogenic mutations in a panel of 52 CH-associated genes with a frequency of at least 2%.

Marshall et al [1] analyzed blood DNA sequencing data from almost 25 thousand cancer cases. They investigated five different cancer predisposition genes to find the cases that could be linked to hereditary forms of breast, ovarian, prostate or pancreas cancer. BRCA1, BRCA2, and PALB2 have a central role in the homologous recombination pathway of DNA double strand break repair. If the proteins encoded by these genes do not function properly, HR is not initiated, and increased levels of chromosomal instability can be observed. In addition, tumors caused by mutations in one of these genes show increased levels of point mutations in a characteristic pattern, called a mutational signature [5]. It is not completely clear how the gene defect causes these point mutations, but the pattern has been observed consistently in these tumors.

In addition to these core DNA repair genes, they also analyzed ATM (which is involved in multiple DDR processes, including DNA double strand break repair, cell cycle regulation, and apoptosis induction), and CHK2 (which is mainly involved in G2/M cell cycle checkpoint regulation). They investigated whether cancer patients with germline mutations in one of these five genes had an increased chance of developing CH. 

Although the question appears straightforward, interpretation of the data is not directly obvious. They found increased CH formation in breast cancer cases with DDR gene mutations and not in the other cancer types studied. At first sight that was unexpected, as CH formation should not be dependent on the type of cancer developed in the patient. However, statistics are not always easily interpretable. For example, CH increases with age, which means that the background level of CH is much higher in older patients. This could be an explanation for the lack of effects in prostate cancer patients, who are generally much older than other cancer patients. Although the authors corrected for age, this may influence the significance of differences, as the background levels are much higher in older patients. 

Furthermore, the number of patients with DNA repair gene mutations contributes to the level of significance that can be reached. The relative risk for breast and ovarian cancer patients was similar (1.42 for breast cancer and 1.32 for ovarian cancer), but the result did not reach significance for ovarian cancer, possibly due to the lower number of patients in this group.

Finally, not all patients received the same treatment. Most breast and ovarian cancer patients had cytotoxic chemotherapy and/or radiotherapy in their treatment schedule. These treatments are known to induce CH [4]. Therefore, the increased CH in these patients may be the result of treatment rather than repair gene status. However, it can be expected that most patients without germline repair gene mutations also received similar treatments, suggesting that the CH is at least influenced by genetic predisposition. However, increased CH may very well be the result of a combination of less efficient DNA repair and increased levels of DNA damage caused by the treatment. 

The numbers of patients in this study were not sufficient to stratify the group for all these parameters, but a more targeted investigation into the possible link between genetic predisposition and treatment with CH would be very interesting as a follow up to this study. This is especially important when we consider the use of specific DNA repair inhibitors, such as PARP inhibitors for breast and ovarian cancer with BRCA1 or BRCA2 defects. More extensive investigation will be required to pinpoint how genetic predisposition and treatment modalities interact to induce CH and whether this is a concern for these patients (will CH eventually develop into leukemia or myelodysplastic syndrome?). 

Another issue is the heterozygous state of the DDR gene mutations in these patients. In the tumors, the wild type allele is lost due to genetic or epigenetic mechanisms, but this is probably not the case in the white blood cells that give rise to CH. It is currently not clear whether mutations in the five genes studied might show haploinsufficiency, meaning that their function is already reduced in the heterozygous situation. It is also possible that cells go through transient periods of inactivation of the functional allele, resulting in vulnerable periods to acquire the additional mutations leading to growth advantage and CH development. All these issues would benefit from in depth mechanistic analysis that will help understand the various parameters contributing to developing CH and secondary malignancies.

Novel treatment options that target the DDR have already been incorporated into clinical practice (PARP inhibitors) or are being tested in clinical trials (e.g. ATR inhibitors) [6]). Therefore, it is highly important to determine how such treatments would influence chances to develop secondary malignancies, especially when such treatments move to earlier phases of cancer treatment or become maintenance therapy. In addition, in light of efforts to induce homologous recombination deficiency (e.g., by using high concentrations of curcumin [7]), it will be important to unravel the various factors influencing CH formation. Many patients are cured or have a relatively long life expectancy with cancer today, raising the chances of developing secondary malignancies, such as hematologic cancers. This makes a deeper understanding of the influence of genetic predisposition and anti-cancer treatment on CH development a prerequisite to optimize therapeutic interventions with minimal risks for developing secondary malignancies. Further studies should specify which genetic markers and which treatments contribute to these possibly deleterious effects of treating the primary cancer.

## Figures and Tables

**Figure 1:**
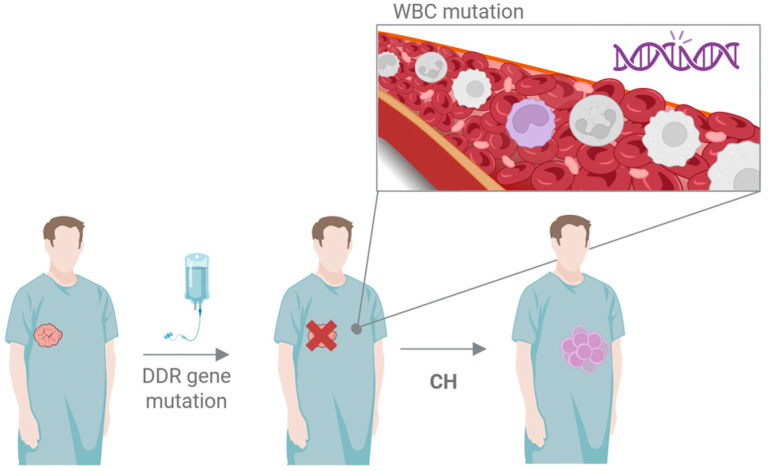
Hypothetical sequence of events for the induction of clonal hematopoiesis (CH): treatment of the original tumor can lead to mutations in a white blood cell (WBC) precursor that can then grow to form a clone with a mutation in a cancer driver gene. This chance may be enhanced in patients with a DDR gene mutation upon antitumor treatment.
